# Supine Versus Prone Percutaneous Nephrolithotomy (PCNL): A Single Surgeon’s Experience

**DOI:** 10.7759/cureus.41944

**Published:** 2023-07-16

**Authors:** Deerush Kannan, Mohsin Quadri, Praveen G Sekaran, Rajesh Paul, Aarthy Panneerselvam, Nitesh Jain

**Affiliations:** 1 Urology, Apollo Hospitals, Chennai, IND; 2 Urology, Guntur Medical College Hospital, Guntur, IND; 3 General Surgery, Saveetha Medical College and Hospital, Chennai, IND

**Keywords:** patient outcomes, prone pcnl, prone positioning, supine pcnl, supine position, percutaneous nephrolithotomy (pcnl), percutaneous renal surgery

## Abstract

Introduction: Percutaneous nephrolithotomy (PCNL) is one of the greatest advances in the field of urology and has been considered the gold standard in the treatment of renal calculi of more than 2 cm in size. While both the supine and prone positions offer their unique advantages, it is still being debated which position offers the most in terms of surgical outcomes. We have evaluated the two approaches in terms of operative time, success rate, stone clearance rate, safety, and complications.

Methods: This prospective cohort study was done in the urology department of a tertiary care center in South India between January 2018 and October 2020. A total of 166 patients, with 83 in supine and 83 in prone positions, were included in the study.

Results: Both groups were matched in terms of age, body mass index, stone size and location, co-morbidities, medications taken, presence of diverticular stone, history of surgery, and baseline creatinine level. Mean operative time and pain scores were noted to be less in supine position as compared to prone. Ease of puncture was superior in supine position. Stone residue was noted to be higher in supine PCNL as well.

Conclusion: Supine PCNLs are preferred in high-risk patients while the prone position is preferred in bilateral PCNLs, complex anatomy, or larger stone burden.

## Introduction

Percutaneous renal surgery is considered one of the greatest advances in the field of minimally invasive urologic procedures. The goal of percutaneous nephrolithotomy (PCNL) is to achieve a complete stone clearance as well as to control pain and to have an uneventful post-operative period [1]. European Association of Urologists (EAU) recommendations state that PCNL is still the gold standard for large, complicated renal stones [2]. Compared to other forms of treatment, the utility of PCNL is unaffected by the size of the stone. The best option for renal stones larger than 20 mm remains PCNL. For stones measuring between 10 and 20 mm, retrograde intrarenal surgery (RIRS) is a viable alternative due to better stone-free rates [2].

First described by Fernstrom and Johansson in 1976, PCNL has typically been performed in patients in the prone position due to a high success rate and low morbidity [3]. The advantages include easier identification of anatomy and better site selection for puncture. It also offers a relatively larger surface area for percutaneous access with a correspondingly lower risk of injury to the abdominal viscera [4]. The primary concern with the prone position is the patient's cardiovascular health, particularly in obese individuals. These shortcomings have contributed to the development of the supine position. Advantages include the greater ease of the anesthesiologist to manage the patient, as there is no need to reposition the patient following the initial insertion of the retrograde ureteric catheter in the supine position thereby reducing cardiovascular and respiratory risks [4]. 

Although there have been numerous studies debating between supine and prone PCNL, there has been no study to date based on a single surgeon's experience. Each surgeon has their preference which may contribute to a bias in the procedures performed. In our study, the surgeon’s track record includes more than 1,000 PCNLs in the prone position and 300 in the supine position. We have evaluated the two approaches in terms of operative time, success rate, stone clearance rate, safety, and complications.

## Materials and methods

This prospective cohort study was done in the urology department of a tertiary care center in South India between January 2018 and October 2020. A total of 166 patients with renal stones underwent PCNL, 83 of them in the supine and 83 in the prone position. Randomization was done on an odd and even basis based on the visit to the outpatient department. Written informed consent for the surgery and the complications and inclusion for study purposes without revealing the identity was taken from all patients priorly as a part of the consent process. Ethical approval was obtained from the institutional ethics committee (approval #ECR/37/Inst/TN/2013/RR-16).

The inclusion criteria were stones of less than 3 cm in maximum diameter, bilateral stones, and previous history of stone disease. Pediatric age group patients (<18 years), patients with active urinary tract infections, and complex anatomy like horseshoe kidney, pelvic kidney, and post-transplant graft kidney were excluded from the study.

Along with standard pre-operative investigations including blood counts and renal function, pre-operative imaging was performed using computed tomography to confirm the diagnosis. Pre-operative urine culture was done in all patients and in case of growth of bacteria, the patient was started on culture-appropriate intravenous antibiotics.

Surgery steps

Prone Position

After ureteral catheterization with the ureteric catheter fixed to an indwelling Foley catheter, the patient is placed in the prone position. A 15 cm long 18 G puncture needle was used for the initial puncture, either in the bull's eye or through triangulation techniques.

A Terumo guide wire measuring 0.035" is passed through the needle and serial dilatation is performed over a metallic guide rod (depending on the decision to deploy a mini or standard nephroscope that was decided based on stone characteristics and calyx size, dilatation was performed up to 15 Fr in cases of mini PCNL and up to 24 Fr in conventional PCNL). Stone was fragmented with a pneumatic lithoclast or thulium laser (2-5 Joule/30-50 Watt setting) and then recovered using an alligator fork. In some cases, additional punctures were required to achieve clearance. In each instance, a double J stent of size 5 Fr was inserted via antegrade fashion after looking for stone clearance intraoperatively.

Supine Position

**Figure 1:**
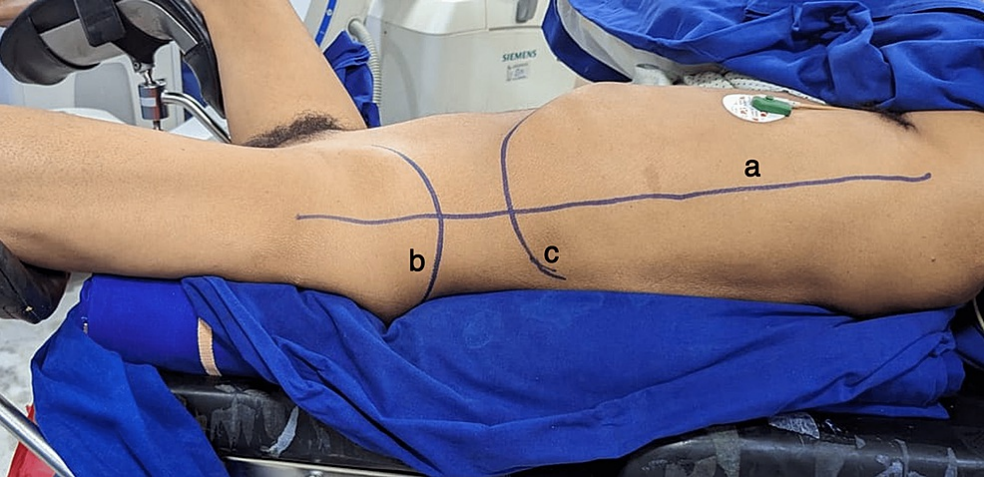
Galdakao-modified supine Valdivia position for percutaneous nephrolithotomy. The image shows (a) posterior axillary line, (b) iliac crest, and (c) 12th rib.

Patients were placed in a Galdakao-modified supine Valdivia position and ureteral catheterization was performed. The ipsilateral leg of the patient is left straight on the stirrup and the contralateral leg is flexed and abducted. The 12th rib, iliac crest, and posterior axillary line were all marked on the skin. Two bolsters are used, one under the hip and the other under the chest, to raise the flank are placed (Figure 1). An 18 cm long 18G needle is used for the initial puncture when using the triangulation procedure. The remainder of the process is identical to that of prone PCNL.

Following surgery, all patients were followed up for immediate and delayed complications. After six weeks of PCNL, an x-ray of the kidney ureter bladder (KUB) was performed to determine whether the patient was stone-free.

The continuous data were presented as mean±SD and categorical data as frequency and percentage. The normality of the data was assessed by the Shapiro-Wilk test. Student's t-test and Mann Whitney U test were used to find out the significant difference between two different surgical postures. Chi-square and Fisher's exact tests were used to find out the association between patients' details and surgical posture. Paired t-test was used to assess the difference in hemoglobin levels before and post-procedure. A p-value of <0.05 was considered statistically significant. All analyses were carried out by using SPSS software version 28.0 (Armonk, NY: IBM Corp.).

## Results

The number of male and females in each group were nearly the same with 50 men in the supine group and 48 men in the prone group. The supine PCNL group had a mean age of 45.7 years while the prone PCNL group had a mean age of 46 years, which was not statistically significant. The mean body mass index (BMI) was also comparable among the two groups with 22.9 in the supine and 23.1 in the prone group (Table 1). The groups were comparable in terms of comorbidities assessed according to the American Society of Anesthesiology (ASA) grading. Thirteen patients in the supine group and 14 patients in the prone group were on antiplatelets which was not significant.

**Table 1:** Demographic characteristics of the patients. *Student’s t-test/Mann-Whitney U test. **Chi-square test/Fisher’s exact test.

Parameters		Surgical posture, n (%)		p-Value
		Supine (n=83)	Prone (n=83)	
Age (years)		45.7±11.9	46.0±11.2	0.862*
Gender	Male	50 (60.2)	48 (57.8)	0.752**
	Female	33 (39.8)	35 (42.2)	
BMI		22.9±2.2	23.1±2.1	0.595*

**Figure 2:**
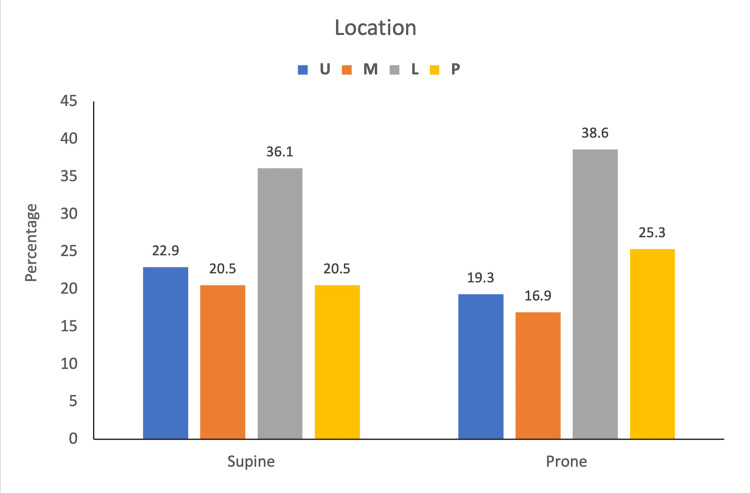
Location of calculus in patients. U: upper calyx; M: middle calyx; L: lower calyx; P: renal pelvis

The location and size of the calculus (1.6-3 cm) among the groups were not significantly different (Figure 2). The most common location was lower calyx. Seven patients in the supine group and eight patients in the prone group had diverticular stones. There were eight patients with bilateral stone disease of which four were performed in the prone position and four in supine. The mean creatinine value in both groups did not have a significant difference. Four patients in the supine group and five patients in the prone group were recurrent stone formers who had previously undergone pyelolithotomy (Table 2).

**Table 2:** Pre-operative factors in patients. *Chi-square test/Fisher’s exact test. **Student’s t-test/Mann-Whitney U test. P-value<0.05 indicates statistical significance. ASA: American Society of Anesthesiologists; U: upper calyx; M: middle calyx; L: lower calyx; P: renal pelvis

Parameters		Surgical posture, n (%)		p-Value
		Supine (n=83)	Prone (n=83)	
ASA	1	46 (55.4)	46 (55.4)	>0.99*
	2	36 (43.4)	36 (43.4)	
	3	1 (1.2)	1 (1.2)	
Antiplatelets		13 (15.6)	14 (16.8)	0.83*
Stone Size		2.4±0.4	2.4±0.4	0.81**
Location	U	19 (22.9)	16 (19.3)	0.79*
	M	17 (20.5)	14 (16.9)	
	L	30 (36.1)	32 (38.6)	
	P	17 (20.5)	21 (25.3)	
Diverticular stone		7 (8.43)	8 (9.63)	0.78*
History of pyelolithotomy		4 (4.81)	5(6.02)	0.73*
Creatinine		1.1±0.5	1.1±0.5	0.92**

In our study, we have determined that the ease of puncture was significantly easier in a prone position than supine (p<0.05), especially when bull’s eye technique was used. Ease of puncture was the number of attempts to make a single access to the pelvicalyceal system and the number of additional accesses required for stone clearance is denoted as additional punctures. The sheath size used for stone clearance and the use of laser/lithoclast was similar among both groups. Once the surgeon felt that the stone was cleared from the calyx, residual stones were screened using fluoroscopy and nephroscopy before proceeding with stenting. Significantly more patients had a stone residue in supine than in prone (p<0.001).

While each group had five patients in which additional puncture was needed to clear the stone fragment, a significant difference was noted in the need for further dilatation in the supine group. Two patients in the supine group were directly taken for endoscopic combined intrarenal surgery (ECIRS) due to complex anatomy which made any efforts of stone-guided puncture in supine position futile. In cases where the lower ureter was tight, ECIRS was not an option and an additional puncture had to be done to clear the stone. Also in two patients, ECIRS could not be successfully performed due to poor vision secondary to the clots due to PCNL. All the study participants were stented with a double J stent as a part of the institute protocol. Percutaneous nephrostomy (PCN) tube was kept in situ in patients where post-operative bleeding was highly possible due to intraoperative difficulty. The number remained the same in both groups.

Mean operative time was significantly less in the supine position (p<0.001). However, we found the mean time from the attempt of initial puncture to the completion of the procedure was 32.2 minutes and 28.4 minutes in the supine and prone groups, respectively (Table 3).

**Table 3:** Intraoperative factors in patients. *Chi-square test/Fisher’s exact test. **Student’s t-test/Mann-Whitney U test. P-value<0.05 indicates statistical significance. PCN: percutaneous nephrostomy

Parameters		Surgical posture, n (%)		p-Value
		Supine (n=83)	Prone (n=83)	
Number of punctures	1	72 (86.7)	74 (89.2)	0.214*
	2	8 (9.6)	9 (10.8)	
	3	3 (3.6)	0 (0)	
Sheath Size		17.4±2.9	17.4±2.8	0.963**
Procedures	Laser	48 (57.8)	48 (57.8)	>0.99*
	Lithoclast	35 (42.2)	35 (42.2)	
Residue		32 (38.5)	12 (14.5)	<0.001*
Actual residue		11 (13.3)	6 (7.2)	0.201*
Additional procedures	Additional puncture	5 (6.0)	5 (6.0)	0.395*
	Dilatation	4 (4.8)	1 (1.2)	
PCN		9 (10.8)	9 (10.8)	>0.99*
Time from induction		45.1±11.2	57.7±11.2	<0.001**
Time from Position		32.2±10.9	28.4±9.0	0.032**

In the supine group, 67 patients had post-operative pain scores more than 5 on the visual analog scale and 16 patients had pain of less than 5, whereas in the prone group, 76 had a score more than 5 and seven patients had a score less than 5. Post-operative fever was seen in two patients in the supine group and one patient in the prone PCNL group. The duration of hospital stay was mostly a day in both groups with 75 patients in supine and 71 patients in the prone group having a hospital stay of one day. The fall in hemoglobin was 0.5 g/dL in the supine group and 0.4 g/dL in the prone group which was not significant. Two patients in the supine group and one in the prone had the late complication of pseudoaneurysm that was managed by angioembolization.

On the sixth week follow-up with imaging on x-ray KUB, seven patients in the supine group and two patients in the prone group had residual stones. This was not significant. As the residual stone sizes were between 4 and 6 mm, patients were managed by retaining the stents for four more weeks and followed up. In this subgroup of patients with residual stones at six weeks follow-up, two in the supine PCNL group required ESWL on further follow-up. The remaining patients showed clearance of stones in four weeks (Table 4).

**Table 4:** Post-operative factors in patients. *Chi-square test/Fisher’s exact test. P-value<0.05 indicates statistical significance.

Parameters		Surgical posture, n (%)		p-Value
		Supine (n=83)	Prone (n=83)	
Fever		2 (2.4)	1 (1.2)	0.56*
Pain score (visual analog scale)	≥5	67 (80.7)	76 (91.6)	0.04*
	<5	16 (19.3)	7 (8.4)	0.04*
Duration of stay (days)	1	75 (90.4)	71 (85.5)	0.34*
	2	8 (9.6)	11 (13.3)	0.46*
	3	0 (0)	1 (1.2)	>0.05*
Late complications		2 (2.4)	1 (1.2)	0.56*
Residue at six weeks		7 (8.4)	2 (2.4)	0.08*

## Discussion

Percutaneous nephrolithotomy (PCNL) has stood the test of time as being the gold standard treatment for large renal calculi, usually defined as >2 cm [5]. Even in smaller stones, it remains a viable alternative. In our study, we have compared outcomes in patients undergoing PCNL for renal stones in supine and prone positions.

Previous studies have demonstrated the superiority of supine PCNL, owing to its shorter operative time and anesthetic advantages [6]. However, these studies excluded patients with complicated stone disease. This demonstrates the need for the prone PCNL. There does remain a certain population of urologists who despite being proficient in prone PCNL desire to acquire newer and updated techniques of supine PCNL as well. 

Patients with a previous history of surgery for stone disease demonstrated a shorter operative time in the prone position. This was also observed in those with diverticular stones or stones which appeared to lie outside of the collecting system in pre-operative imaging. In our study, the bull's eye technique has proved superior in such scenarios with better access, stone clearance rate, and a lesser number of punctures. In a study on management of calyceal stones by Patodia et al., they concluded that stone-guided puncture without dilation or creation of neo-infundibulum reduced operative time and morbidity with higher stone-free rate [7]. As compared to the supine, the prone position has a significantly shorter nephrostomy tract length and more potential access sites that provides ease and safety of percutaneous renal access [8].

In the absence of such adverse factors, our study reported a significant decrease in the mean operative time of supine PCNL compared to prone (p<0.001). This has been noted in other studies as well. It is interesting to note that the actual procedure time is essentially the same, while it is the pre-puncture time that is increased in prone PCNL [6,9,10]. This can be ascribed to the time taken for positioning the patient in the prone position. If there is stone migration in supine PCNL, the operative time is again prolonged. This can be due to the need to transfer to ECIRS or the need for additional punctures.

Our study reports the ease of puncture, taken as the number of punctures needed for access, to be easier in prone than supine. No bowel injury was reported in our study. Other studies have reported a 0.2-0.3% incidence of colonic perforation, although it was noted chiefly in cases of complex anatomy like horseshoe kidney which was excluded in our study [11,12].

**Figure 3:**
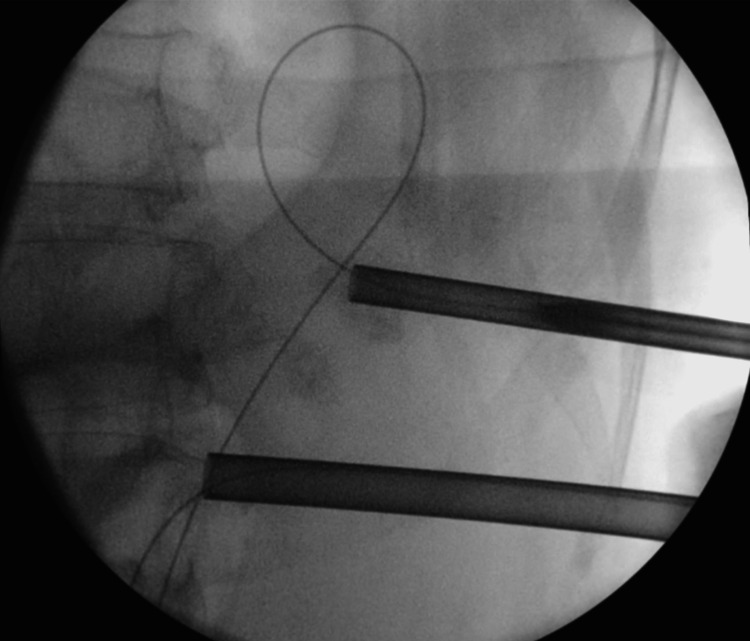
The additional puncture was made to reach the upper calyx in a patient with lower calyceal stone who had stone migration during supine PCNL. PCNL: percutaneous nephrolithotomy

Stone migration in supine PCNL has been noted to be higher among stones in the middle or lower calyces (Figure 3) [13]. These patients require the use of excessive torquing of nephroscope, additional punctures, or ECIRS to achieve stone clearance. The difficulty to access the upper-pole calyx in a supine position was attributed to the posteromedial location as well as its position within the ribs [14].

These techniques to clear the migrated fragments have been associated with a higher risk of bleeding [15,16]. ECIRS is a well-known advantage in supine PCNL [12]. The question of whether higher stone migration rates in supine PCNL are the reason for ECIRS becoming more prevalent remains unanswered. We have also observed that stone migration rates were higher in the supine PCNL group. In contrast, however, our study noted that none of the patients in the prone PCNL groups required ESWL on follow-up as compared to the two patients in the supine PCNL group.

In institutes performing PCNL as day-care procedures, post-operative pain score remains an integral part of the discharge criteria [17]. Our study reports lower pain scores in supine than prone PCNL. Various reasons, such as residual stones and operative time, have been postulated as the cause [18]. We also noted a lower operative time but a higher rate of residual stones in the supine PCNL group. These results suggest the successful possibility of PCNL in either position as a daycare procedure if all the discharge criteria are met.

Complication rates of both early and late complications, such as post-operative fever or hematuria, were slightly higher in the supine group. The fall in post-operative hemoglobin was not significant in our study. Compared to the study by Ketsuwan et al. which reported a blood transfusion rate of 9.29%, our study participants didn't require any blood transfusions [19].

Some limitations in our study were that sub-grouping analysis on diverticular stones and previously operated patients were not done as the numbers in this sub-group were small. Follow-up for stone clearance was done with x-ray KUB and not computed tomography considering the radiation exposure involved to the patients.

## Conclusions

While there is no clear distinction regarding the superiority of one position over the others, patient factors and surgeon preference should dictate the mode of management. Supine PCNLs are preferred in high-risk patients. For scenarios like bilateral PCNL where operative time gets increased in positioning the patient in supine or complex anatomy of the collecting system, large stone burden requiring more than two punctures, or diverticular stones which require the bull’s eye technique, prone PCNL is preferred.
